# Association of d-dimer levels with in-hospital outcomes among COVID-19 positive patients: a developing country multicenter retrospective cohort

**DOI:** 10.1097/MS9.0000000000000633

**Published:** 2023-04-13

**Authors:** Muhammad Junaid Tahir, Farah Yasmin, Unaiza Naeem, Hala Najeeb, Kamlesh Kumar, Arti, Rahul Robaish Kumar, Rahul Robaish Kumar, Abdul Majeed, Rahul Kumar, Agha Wali, Sandhya, Ramsha Shahab, Ramsha Shahab, Moustafa Hegazi, Khabab Abbasher Hussien Mohamed Ahmed, Muhammad Sohaib Asghar

**Affiliations:** a Department of Radiology, Pakistan Kidney and Liver Institute and Research Center, Lahore, Pakistan; b Department of Internal Medicine, Dow Medical College, Dow University of Health Sciences, Karachi, Pakistan; c Department of Internal Medicine, Jinnah Sindh Medical University, Karachi, Pakistan; d Department of Internal Medicine, Liaquat College of Medicine and Dentistry, Karachi, Pakistan; e Department of Internal Medicine, Jinnah Postgraduate Medical Center, Karachi, Pakistan; f Department of Internal Medicine, Dow University Hospital–Ojha Campus, Karachi, Pakistan; g Chandka Medical College, Larkana, Pakistan; h Department of Internal Medicine, Ghulam Muhammad Mahar Medical College Sukkur, Pakistan; i Division of Pulmonology and Critical Care, Mayo Clinic, Rochester, USA; j Faculty of Medicine, University of Khartoum, Khartoum, Sudan; k Bolan Medical Complex Hospital, Quetta, Pakistan

**Keywords:** coagulopathy, d-dimer, hospital mortality, ICU, SARS-CoV-2, severity

## Abstract

D-dimer levels, which originate from the lysis of cross-linked fibrin, are serially measured during coronavirus disease 2019 illness to rule out hypercoagulability as well as a septic marker.

**Methods:**

This multicenter retrospective study was carried out in two tertiary care hospitals in Karachi, Pakistan. The study included adult patients admitted with a laboratory-confirmed coronavirus disease 2019 infection, with at least one measured d-dimer within 24 h following admission. Discharged patients were compared with the mortality group for survival analysis.

**Results:**

The study population of 813 patients had 68.5% males, with a median age of 57.0 years and 14.0 days of illness. The largest d-dimer elevation was between 0.51–2.00 mcg/ml (tertile 2) observed in 332 patients (40.8%), followed by 236 patients (29.2%) having values greater than 5.00 mcg/ml (tertile 4). Within 45 days of hospital stay, 230 patients (28.3%) died, with the majority in the ICU (53.9%). On multivariable logistic regression between d-dimer and mortality, the unadjusted (Model 1) had a higher d-dimer category (tertile 3 and tertile 4) associated with a higher risk of death (OR: 2.15; 95% CI: 1.02–4.54, *P*=0.044) and (OR: 4.74; 95% CI: 2.38–9.46, *P*<0.001). Adjustment for age, sex, and BMI (Model 2) yields only tertile 4 being significant (OR: 4.27; 95% CI: 2.06–8.86, *P*<0.001).

**Conclusion:**

Higher d-dimer levels were independently associated with a high risk of mortality. The added value of d-dimer in risk stratifying patients for mortality was not affected by invasive ventilation, ICU stays, length of hospital stays, or comorbidities.

## Introduction

Highlights

- This multicenter retrospective observational study was carried out in two tertiary care hospitals.
- Higher d-dimer levels were independently associated with a high risk of mortality.
- With the use of anticoagulation to reduce mortality in coronavirus disease 2019 patients, the ability of d-dimer as a risk marker for mortality is further justified in hypercoagulable states.

The burden of cardiovascular and thrombotic events in patients with coronavirus disease 2019 (COVID-19) is a growing concern. Reports show manifestations of macro- and micro-vascular thrombotic events in severe to critically ill COVID-19 patients^1^. The hypercoagulable and inflammatory state found in COVID-19 patients is likely due to activated endothelial cells^1^. In response to this prothrombotic phenomenon, the levels of plasma d-dimer are elevated^2^,^3^,^4^, and similar findings are reported in the coagulopathy phenomenon in patients with COVID-19^5^.

D-dimer is a fibrin degradation product and is established as a venous thromboembolism risk factor^6^. The decomposition of cross-linked fibrins during the process of coagulopathy results in the formation of d-dimer, a small fragment protein^7^. D-dimer is an indicator of fibrin turnover, fibrinolysis, and intravascular thrombosis^8^,^9^. It is also elevated in a variety of pathological conditions, including disseminated intravascular coagulopathy (DIC), pulmonary embolism, deep venous thrombosis, sepsis, cancer, previous trauma, previous surgical intervention, cerebrovascular stroke, and ischemic heart disease^7^,^9^,^10^.

D-dimer is not an exclusive indicator of sepsis, since the role of presepsin, procalcitonin, C-reactive protein, interleukin 6, and other biomarkers have not been correlated. D-dimer is regarded as an effective clinical marker in identifying the disease severity and respiratory distress in the COVID-19 patient population as thrombosis is suspected to cause disease severity and respiratory distress in these patients^6^,^11^,^12^. D-dimer is associated with the degree and prognosis of lung injury. Fibrin formed locally in the lungs degrades to form d-dimer, due to lung injury, and d-dimer is then collected in the alveoli and lung parenchyma and consequently moves to the bloodstream^5^,^13^. A local pulmonary thrombosis is an immune-hemostatic response to limit further spread of the virus, and a breakdown of these microthrombi leads to elevated levels of d-dimer. This poses a risk of increased disease severity and mortality^14^.

Changes in d-dimer levels in hospitalized patients with COVID-19 for the assessment of outcomes have been assessed in several studies^15^. In an observational study from China, the mean d-dimer concentration was significantly higher in nonsurvivors compared with survivors^16^. Similarly, another retrospective Chinese study showed elevated levels of d-dimer (5.35 mg/l vs. 0.98 mg/l) in deceased patients as compared to the survivors of COVID-19^17^. Several studies have corroborated that elevated levels of d-dimer levels are associated with disease severity and mortality rates^6^,^9^. However, there have been contradicting findings too. Premkumar *et al*.^18^ reported that d-dimer and normalized d-dimer were not predictive of venous thromboembolism or mortality in a prospective study from a tertiary care center that enrolled COVID-19 pneumonia patients requiring ICU admission. However, their study has significant selection bias as they chose only ICU admission patients and only ones with complete data reducing the patient sample size to 74. Despite this patient on mechanical ventilation compared to low flow oxygen did approach significance for d-dimer levels.

Nonetheless, elevated levels of d-dimer have been appreciably detected in COVID-19 patients^19^ and present circumstances warrant that laboratory parameters that are predictors of mortality and disease progression are identified. D-dimer levels are a probable hallmark of COVID-19 severity^15^ and in our study; we have monitored d-dimer levels with respect to clinical outcomes in adult COVID-19 patients and assessed its utility as a prognostic marker. The hypothesis was to confirm the multifold increase of d-dimer levels being highly associated with mortality.

## Methods

This was a multicenter retrospective study that enrolled consecutive adults with laboratory-confirmed COVID-19 admitted to two tertiary care hospitals of Karachi, Pakistan. Data was collected by the two principal authors by detailed chart review and entered into Microsoft Excel sheets. Both the principal authors validated the data authenticity and consistency through manual checks. Institutional review boards of both hospitals gave the ethical approval and informed consent was taken from every participant/guardian at the admission in the COVID-19 unit as per the institutional regulations. We included patients admitted to health care facilities with COVID-19 infection, with at least one measured level of d-dimer during the hospital stay. D-dimer monitoring is part of routine clinical care in COVID-19, and measurements are serially performed during the course of illness to rule out hypercoagulability as well as sepsis. However, we chose admission day levels for analysis to prevent the influence of treatment. D-dimer levels are tested using 8–15 µl of sample with an immunoturbidimetric assay that had a reference range of 0–0.50 µg/ml (Sysmex®, CS-2500). The manufacturer claims the diagnostic sensitivity of 97.8% with a lower 95% CI of 95.9%. For the analysis, levels of d-dimer were categorized, as guided by local recommendations, into four ranges (tertiles): less than 0.50 µg/ml (reference range), 0.51–2.00 µg/ml, 2.01–5.00 µg/ml, and greater than 5.00 µg/ml. Patients were followed until any clinical outcomes (either discharge from the hospital or death) was achieved. The longest follow-up of an in-hospital stay was 45 days. Patients who were discharged alive from the hospital were considered survivors and those were compared with the mortality group for survival analysis. Exclusion criteria included pregnancy, a known hypercoaugulable state, and previous thrombosis.

Categorical variables were expressed as frequency and percentage, while continuous variables were expressed as median and interquartile range (IQR). Multivariable logistic regression was used to categorize the association of increasing d-dimer levels and 45-day in-hospital mortality. The initial concentration of d-dimer was usually measured within the first 24 h following admission. For adjustment of potential confounders between levels of d-dimer and mortality, Model 1 remained an unadjusted odds ratio (OR). Model 2 was adjusted for age, sex, and BMI. Model 3 was adjusted for comorbidities like diabetes mellitus, hypertension, ischemic heart disease, chronic obstructive pulmonary disease, asthma, chronic kidney disease, and chronic liver disease. Model 4 was further adjusted for mode of mechanical ventilation, ICU stay, length of hospital stay, and treatment modalities like therapeutic anticoagulation, remdesivir, tocilizumab, and steroids during the hospital stay. Kaplan–Meier survival curves were obtained for each of the d-dimer tertiles predicting 45-day in-hospital survival. A log-rank test was carried out to compare the survival distributions and the number of populations at risk. Strengthening the reporting of cohort, cross-sectional and case-control studies in surgery (STROCSS) 2021 guidelines were conformed while drafting the manuscript^20^.

## Results

The study population included 813 patients from two tertiary care centers of Karachi, Pakistan. Of all the enrolled patients, 68.5% were male. The median age of the patient population was 57.0 years and 62.6% of the patients belonged to the age group of 51–75 years. The median BMI was 26.6 kg/m^2^, median duration of illness was 14.0 days, and the median length of hospital stay was 7.0 days. Among the 813 patients with measured d-dimer, those with higher levels were more likely to be older, stayed in ICU, had higher rates of invasive mechanical ventilation (IMV), and were more likely to be diabetic and hypertensive. Patients with higher d-dimer levels were more likely to be receiving therapeutic anticoagulants and intravenous or oral steroids during the hospital stay.

D-dimer levels were elevated in most patients, with only 99 patients (12.2%) having values below the reference range (tertile 1). Patients with d-dimer levels greater than 5.00 mcg/ml were 29.2% of the population (*n*=236) (tertile 4). However, most of the patients, 332 patients (40.8%), had d-dimer levels ranging in 0.51–2.00 µg/ml (tertile 2). Levels of d-dimer in 146 patients (17.9%) were in between 2.01–5.00 µg/ml (tertile 3). Within 45 days of hospital stay, 230 patients (28.3%) died, with the majority staying in the ICU (53.9%). Surviving 583 (71.7%) patients were discharged and could not be followed up for extended periods to assess long-term mortality rates. The rate of mortality in patients in tertile 1 was lower than the patients in tertile 4 (15.1 vs. 44.1%), as shown in Table 1. Similarly, most of the patients in tertile 1 did not need ventilation support (37.4%) whereas 27.1% of patients in tertile 4 were invasively ventilated.

**Table 1:** Baseline and clinical characteristics of the study population according to different d-dimer tertiles (*n*=813)

Variables	All patients (*n*=813)	Tertile 1 (*n*=99)	Tertile 2 (*n*=332)	Tertile 3 (*n*=146)	Tertile 4 (*n*=236)
Median (IQR) Age (years)	57.0 (48.0–67.0)	52.0 (45.0–58.0)	60.0 (49.0–68.0)	61.0 (50.0–70.0)	60.0 (50.0–65.0)
Age groups,*n* (%)					
<25 years	21 (2.6)	3 (3.0)	3 (0.1)	10 (6.8)	5 (2.1)
26–50 years	226 (27.8)	48 (48.5)	91 (27.4)	28 (19.2)	59 (25.0)
51–75 years	509 (62.6)	47 (47.5)	208 (62.6)	97 (66.4)	157 (66.5)
>75 years	57 (7.0)	1 (0.1)	30 (9.0)	11 (7.5)	15 (6.3)
Gender,*n* (%)					
Male	557 (68.5)	71 (71.7)	229 (69.0)	96 (65.7)	161 (68.2)
Female	256 (31.5)	28 (28.3)	103 (31.0)	50 (34.2)	75 (31.8)
Median (IQR) BMI (kg/m^2^)	26.6 (24.2–30.9)	26.6 (24.6–31.1)	26.4 (24.2–30.9)	26.6 (24.6–30.9)	26.2 (24.2–30.4)
BMI groups,*n*(%)					
<26 kg/m^2^	357 (43.9)	53 (53.5)	133 (40.1)	58 (39.7)	113 (47.9)
>26 kg/m^2^	456 (56.1)	46 (46.5)	199 (59.9)	88 (60.3)	123 (52.1)
Median (IQR) Duration of illness (days)	14.0 (10–17.50)	12.5 (9.2–15.2)	15.0 (11.0–18.2)	14.0 (8.5–16.5)	14.0 (10.0–20.0)
Median (IQR) Length of hospital stay (days)	7.0 (4.0–10.0)	6.0 (4.0–10.0)	7.0 (4.0–11.0)	7.0 (4.0–10.0)	6.0 (3.5–10.0)
Hospital stay,*n*(%)					
Isolation ward	375 (46.1)	61 (61.6)	163 (49.1)	60 (41.1)	91 (38.6)
ICU/HDU	438 (53.9)	38 (38.4)	169 (50.9)	86 (58.9)	145 (61.4)
Mortality,*n* (%)	230 (28.3)	15 (15.1)	71 (21.4)	40 (27.4)	104 (44.1)
Median (IQR) d-dimer level (µg/ml)	1.79 (0.80–6.60)	0.35 (0.26–0.44)	0.99 (0.72–1.31)	3.46 (2.68–4.15)	10.79 (7.81–20.00)
Mode of ventilation,*n* (%)					
Invasive	135 (16.6)	11 (11.1)	37 (11.1)	23 (15.8)	64 (27.1)
BiPAP/CPAP	165 (20.3)	13 (13.1)	72 (21.7)	32 (21.9)	48 (20.3)
Oxygen by mask	206 (25.3)	21 (21.2)	89 (26.8)	44 (30.1)	52 (22.0)
Nasal cannula	94 (11.6)	17 (17.2)	33 (9.9)	9 (6.1)	35 (14.8)
None	213 (26.2)	37 (37.4)	101 (30.4)	38 (26.0)	37 (15.7)
Comorbidities,*n* (%)					
Diabetes	330 (40.6)	27 (27.3)	136 (41.0)	63 (43.1)	104 (44.1)
Hypertension	384 (47.2)	44 (44.4)	156 (47.0)	61 (41.8)	123 (52.1)
IHD	87 (10.7)	19 (19.2)	43 (13.0)	15 (10.3)	10 (4.2)
CKD	76 (9.3)	4 (4.0)	25 (7.5)	17 (11.6)	30 (12.7)
COPD/Asthma	61 (7.5)	9 (9.1)	31 (9.3)	10 (6.8)	11 (4.2)
CLD	36 (4.4)	8 (8.1)	12 (3.6)	7 (4.8)	9 (3.8)
Treatment modalities,*n* (%)					
Anticoagulation	461 (56.7)	24 (24.2)	141 (42.5)	107 (73.3)	189 (80.1)
Steroids	654 (80.4)	55 (55.5)	254 (76.5)	121 (82.9)	224 (94.9)
Remdesivir	200 (24.6)	13 (13.1)	63 (19.0)	79 (54.1)	45 (19.1)
Tocilizumab	91 (11.2)	1 (1.0)	10 (3.0)	31 (21.2)	49 (20.8)

Tertile 1: <0.5 µg/ml; Tertile 2: 0.5–2.0 µg/ml; Tertile 3: 2.0–5.0 µg/ml; Tertile 4: greater than 5.0 µg/ml.

Data presented as either median (IQR) or Frequency (%).

BiPAP, bi-level positive airway pressure; CPAP, continuous positive airway pressure; CKD, chronic kidney disease; COPD, chronic obstructive pulmonary disease; CLD, chronic liver disease; HDU, high dependency unit; IQR, interquartile range; IHD, ischemic heart disease; n: number in total.

**Figure 1:**
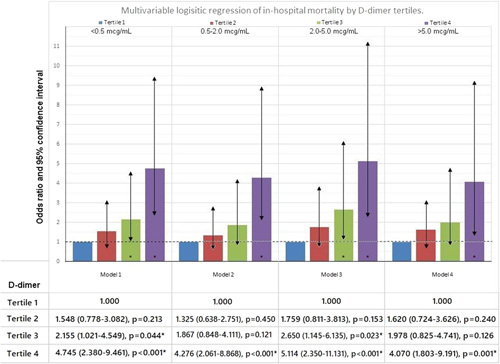
Logistic regression models for 45-day in-hospital mortality by d-dimer tertiles. Model 1 is unadjusted odds ratio. Model 2 is adjusted for age, sex, and BMI. Model 3 is further adjusted for patient characteristics like smoking and comorbidities like diabetes mellitus, hypertension, ischemic heart disease, chronic obstructive pulmonary disease/asthma, chronic kidney disease, and chronic liver disease. Model 4 is further adjusted for mode of mechanical ventilation, ICU stay, length of hospital stay, and treatment modalities like therapeutic anticoagulation, remdesivir, tocilizumab, and steroids during hospital stay.

The correlation between levels of d-dimer and mortality were assessed using multivariable logistic regression. The unadjusted Model 1 had a higher d-dimer category [tertile 3 (OR: 2.15; 95% CI: 1.02–4.54, *P*=0.044) and tertile 4 (OR: 4.74; 95% CI: 2.38–9.46, *P*<0.001)] associated with a higher risk of death. Adjustment for age, sex, and BMI (Model 2) demonstrated a significant correlation of tertile 4 with the higher risk of mortality (OR: 4.27; 95% CI: 2.06–8.86, *P*<0.001). Additional adjustment for comorbidities (Model 3) further showed significant associations of tertile 3 (OR: 2.65; 95% CI: 1.14–6.13, *P*=0.023) and tertile 4 (OR: 5.11; 95% CI: 2.35–11.13, *P*<0.001) with the risk of mortality. Further adjustment for ICU stay, length of hospital stay, invasive ventilation, and treatment modalities including therapeutic anticoagulation (Model 4) only gave a significant relationship of mortality with tertile 4 (OR: 4.07; 95% CI: 1.80–9.19, *P*=0.001), as shown in Figure 1.

**Figure 2:**
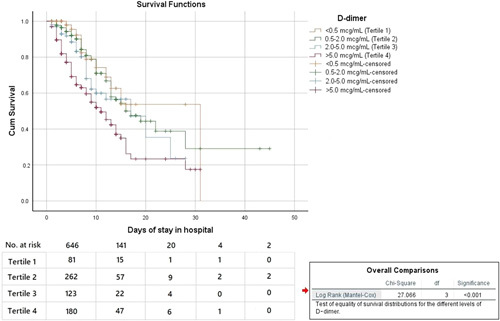
Kaplan–Meier Survival curve for 45-day in-hospital mortality by d-dimer tertiles. A significant difference was observed when comparing surviving/discharged alive patients (*n*=583, 71.7%) with deceased patients (*χ* ^2^: 27.066, degrees of freedom: 3; *P*<0.001).

Results of Kaplan–Meier survival analysis examining the relationship between tertiles of levels of d-dimer and 45-day in-hospital mortality is shown in Figure 2. A significant difference was observed when compared to survivors (*n*=583, 71.7%) and deceased patients (*χ* ^2^: 27.066, degree of freedom: 3; *P*<0.001).

## Discussion

Several studies have reported the hypercoagulable state in patients with severe COVID-19^21^. Later stages of novel coronavirus pneumonia are often associated with a common coagulation and secondary hyperfibrinolysis pathway that leads to moderately or markedly elevated levels of fibrin-related markers such as d-dimer and fibrin degradation product in all deaths^22^. Once inside the host cells, the virus multiplies and eventually infects adjacent cells as the cell bursts. The ACE-2 receptor is expressed on epithelial cells of the gastrointestinal tract, kidneys, vascular endothelial cells, and adipose tissues among many other organs, and this vast array of distribution leads to profuse clinical signs and symptoms. In the severe stages of the disease, high levels of circulating cytokines and chemokines augment the viral process of pneumonia, acute respiratory distress syndrome, sepsis, coagulopathy, and other severe complications of COVID-19^23^. The treatment of choice has usually been corticosteroids, to reduce lung inflammation but recent evidence implies that it delays viral clearance with no effect on mortality but depends on the severity of the disease^24^. D-dimer is a coagulation parameter that can help in detecting early DIC, indicating a hypercoagulable state, and a high risk of thrombosis^21^. Postmortem studies reveal d-dimer elevation has been linked to coagulopathy and with DIC, developing into capillary microthrombosis^23^. Patients with COVID-19 and comorbidities spend a longer duration in an immobile state and are more likely to undergo an invasive procedure leading to the possibility of a thrombotic episode^3^.

D-dimer is an important laboratory marker as d-dimer continuous tendency independently correlates with a worse prognosis in COVID-19 and hence, can be an independent predictor of the short-term prognosis of COVID-19^25^. Additionally, pulmonary embolism can be ruled out in COVID-19 suspected patients based on d-dimer levels^26^. Monitoring d-dimer can be essential when predicting clinical outcomes and expediting recovery in patients afflicted with COVID-19. In our study, 29.2% of patients had the highest d-dimer levels (greater than 5.00 µg/ml). Most patients (40.8%) had the levels of d-dimer between 0.51 and 2.00 µg/ml. The findings of our study are comparable to several other studies. The first study that analyzed the levels of d-dimer levels in patients with COVID-19 showed that d-dimer levels on admission were higher in ICU patients (median d-dimer level: 2.4 mg/l; IQR: 0.6–14.4) than those of the non-ICU patients (median d-dimer level 0.5 mg/l; IQR: 0.3–0.8)^24^. Overall, d-dimer was fivefold higher in those with severe disease^24^. Moreover, Tang *et al*. reported that d-dimer level was 3.5-fold higher in those with severe disease (median: 2.12 mg/l; IQR: 0.77–5.27 mg/l) than in those with nonsevere disease (median: 0.61 mg/l; IQR: 0.35–1.29 mg/l)^22^. Lippi *et al*. in a meta-analysis, delineate that the d-dimer values are considerably higher in COVID-19 patients with severe disease than in those with nonsevere disease (weighed mean difference: 2.97 mg/l; 95% CI: 2.47–3.46 mg/l)^27^. Furthermore, Nugroho *et al*. in another meta-analysis, established that mean d-dimer levels on admission were higher in patients with severe disease than in nonsevere patients^28^. In addition, in a retrospective case study from Pakistan, markedly raised d-dimer levels greater than 550 ng/ml (1624.9 ± 2067.2 ng/ml) were reported in patients admitted with COVID-19 polymerase chain reaction positive result^29^.

Through our results, we concluded that a greater proportion of survivors had d-dimer values less than 0.50 µg/ml (15.1%) compared with nonsurvivors having higher d-dimer values greater than 5.00 mcg/ml (44.1%). This trend resonates with what has been observed in other investigations. In a retrospective study, Wang *et al*. stated that the median d-dimer value in the nonsurvivor group (median: 471ng/ml; IQR: 264–869) was significantly higher than that in the survivor group (median: 184 mg/dl; IQR: 116.5–364)^30^. Moreover, a retrospective study from China showed that d-dimer levels were nearly ninefold higher in patients who died (median: 5.2 mg/l; IQR: 1.5–21.1 mg/l) than in those who survived (median: 0.6 mg/l; IQR: 0.3–1.0 mg/l)^31^. Our results reveal that during the 45 days of hospital stay, 28.3% patients died while the majority (53.9%) stayed in the ICU. Ali *et al*. also suggested that d-dimer levels differ significant between survived and deceased patient groups^32^. Moreover, a meta-analysis revealed that the nonsurvivor group had a higher pooled mean difference of d-dimer levels on admission (MD = 5.54, 95% CI: 3.40–7.67). Litao *et al*. stated that d-dimer levels equal to or more than 2.0 μg/ml was the significant predictor of subsequent deaths and could be used in determining the cutoff value for predicting mortality^3^. Moreover, in another study, Shah *et al*. demonstrated that patients who had d-dimer levels more than 0.5 mg/l had a twofold higher risk of developing a severe case of the disease and a fourfold higher risk of mortality than those who had d-dimer levels less than 0.5 mg/l^33^.

It can be speculated that d-dimer levels may be associated with evolution towards a worse clinical picture^34^, therefore it can be helpful in recognizing disease progression and outcome at an early stage. In a retrospective study from Morocco, a d-dimer level on day 5 greater than 1360 ng/ml was associated with higher odds of in-hospital death, and this was also seen in patients with severe COVID-19 illness. These findings demonstrated that high d-dimer levels over 1360 ng/ml on day 5 should be closely monitored^35^.

Approximately one-fifth of patients with COVID-19 are categorized as seriously or critically ill who develop respiratory failure and require breathing assistance^36^. Respiratory support may be either noninvasive, mainly oxygen support, including high flow systems and noninvasive ventilation, or IMV via tracheal intubation^37^. It is crucial to develop ways to discern which patients require IMV to be initiated at an early stage and which ones might not benefit from noninvasive support^37^. D-dimer levels can plausibly be useful in making this decision. In our study, patients with d-dimer levels less than 0.50 µg/ml, 37.4% patients, did not require ventilation. Additionally, patients with d-dimer levels more than 5.00 µg/ml, 27.4% of patients, were more likely to need invasive ventilation. Zhou *et al*. has reported comparable findings in a meta-analysis that showed mechanical ventilation to be positively associated with d-dimer levels^37^.

Through our regression analysis, we inferred that tertile 4, which corresponded to d-dimer levels greater than 5.00 µg/ml, was significantly associated with mortality across all models in the analysis. Tertile 3, which indicated d-dimer levels between 2.01–5.00 µg/ml, was significantly associated with mortality in the unadjusted analysis (Model 1) and when adjusted for comorbidities (Model 3). Zhou *et al*. in a study from China revealed using multivariable regression model analysis that d-dimer levels greater than 1 μg/ml at admission were associated with increased odds of death^31^. Atalay *et al*. also performed a multivariable regression analysis for predictors of 1-year mortality. Covariates were age, sex, computerized tomography severity score, biomarker concentrations of ferritin, high-sensitivity C-reactive protein, lactate dehydrogenase, cardiac troponin, neutrophil to lymphocyte ratio, uric acid, and d-dimer. Among all the covariates, computerized tomography severity score, sex, and age, d-dimer were the only biomarkers that were associated with mortality^38^. In an Indian study, multivariate analysis showed that age more than 60 years, neutrophil to lymphocyte ratio more than 3.3, and d-dimer more than 1 μg/ml were statistically significant when assessing the odds of getting severe disease in patients with COVID-19^39^. In a meta-regression, Zhao *et al*. concluded that pre-existing medical conditions, including any comorbidity, hypertension, diabetes, chronic lung diseases, and cerebrovascular diseases, were positively associated with d-dimer levels while age, sex, blood pressure, and dyspnea/tachypnea positively correlated with d-dimer. In addition, acute lung/ acute respiratory distress syndrome, heart, kidney, and liver injuries were significantly associated with d-dimer levels. D-dimer was found to be an independent predictor of mortality and was positively associated with mortality^40^. It can be hypothesized that elevated levels of d-dimer is linked to poor outcome in COVID-19 and superimposed co-infection^41^. Higher cutoff values, more than 2 mg/l, are assumed to be better at predicting mortality in COVID-19 with a sensitivity of 92.3% and a specificity of 83.3% after adjusting for age, sex, and comorbidities^3^.

While our findings have strengthened d-dimer’s role in predicting disease prognosis for COVID-19 patients, nevertheless, these may be limited by the retrospective nature of the study. Not compensating for selection bias, and exclusion bias that prevents knowledge of d-dimer in other diseases apart of COVID-19 can be other limiting factors. Serial reporting of d-dimer could be influenced by treatment modalities; hence, we only focus on admission day levels. But, there are many markers of admission mortality for ICU patients, including SOFA and APACHE, however, a lack of other predictor reporting can also limit the generalizability.

## Conclusion

In conclusion, our multicenter, retrospective, observational study of adult COVID-19 patients admitted to tertiary care centers of Karachi, Pakistan, depicted that higher d-dimer levels were independently associated with a high risk of mortality. The added value of d-dimer in risk stratifying patients for mortality was not influenced by invasive ventilation, ICU stay, length of hospital stay, or comorbidities. As recent data seemingly suggests the use of anticoagulation to reduce mortality in COVID-19 patients, the ability of d-dimer as a risk marker for mortality is further justified. Further large-scale prospective studies are required to assess whether therapies modulating the suggested hypercoagulable states in critically ill COVID-19 patients can further reduce the mortality associated with this population.

## Ethical approval

Institutional review boards of Dow University Hospital gave the ethical approval.

## Consent

Written informed consent was taken from the study participants and/or families before inclusion in the study at the time of admission in COVID-19 unit as per the regulations of institution during the pandemic.

## Sources of funding

The authors received no funding for this work.

## Conflicts of interest disclosure

The authors declare that they have no financial conflict of interest with regard to the content of this report.

## Provenance and peer review

Externally peer reviewed, not commissioned.
